# Chromosome-Level Assembly and Comparative Genomic Analysis of *Suillus bovinus* Provides Insights into the Mechanism of Mycorrhizal Symbiosis

**DOI:** 10.3390/jof10030211

**Published:** 2024-03-13

**Authors:** Jinhua Zhang, Mengya An, Yanliu Chen, Shengkun Wang, Junfeng Liang

**Affiliations:** 1 Research Institute of Tropical Forestry, Chinese Academy of Forestry, Guangzhou 510520, China; 2 College of Forestry, Nanjing Forestry University, Nanjing 210037, China

**Keywords:** *Suillus bovinus*, ectomycorrhizal fungi, carbohydrate-active enzymes, secondary metabolism, small secreted proteins

## Abstract

*Suillus bovinus* is a wild edible ectomycorrhizal fungus with important economic and ecological value, which often forms an ectomycorrhiza with pine trees. We know little about the mechanisms associated with the metabolism and symbiosis of *S. bovinus* and its effects on the nutritional value. In this study, the whole-genome sequencing of *S. bovinus* was performed using Illumina, HiFi, and Hi-C technologies, and the sequencing data were subjected to genome assembly, gene prediction, and functional annotation to obtain a high-quality chromosome-level genome of *S. bovinus*. The final assembly of the *S. bovinus* genome includes 12 chromosomes, with a total length of 43.03 Mb, a GC content of 46.58%, and a contig N50 size of 3.78 Mb. A total of 11,199 coding protein sequences were predicted from genome annotation. The *S. bovinus* genome contains a large number of small secreted proteins (SSPs) and genes that encode enzymes related to carbohydrates, as well as genes related to terpenoids, auxin, and lipochitooligosaccharides. These genes may contribute to symbiotic processes. The whole-genome sequencing and genetic information provide a theoretical basis for a deeper understanding of the mechanism of the mycorrhizal symbiosis of *S. bovinus* and can serve as a reference for comparative genomics of ectomycorrhizal fungi.

## 1. Introduction

**Figure 1:**
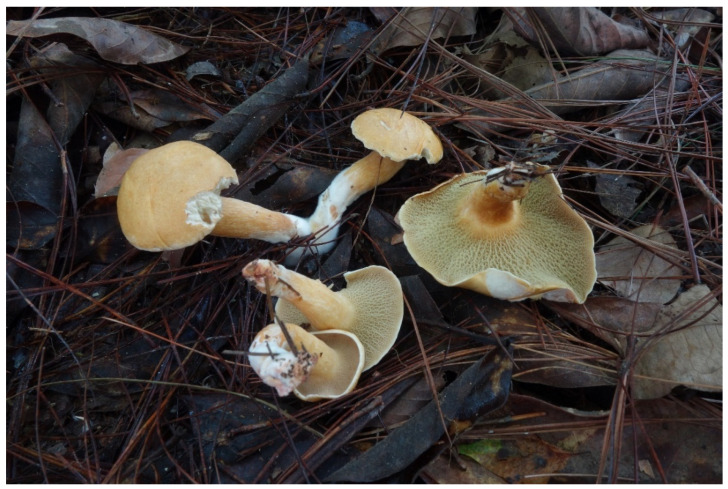
Fruiting bodies of *Suillus bovinus*.

*Suillus bovinus* is a delicious edible ectomycorrhizal (ECM) fungus that belongs to Boletales, Suillaceae, *Suillus* [1]. This fungus often forms mycorrhiza with pinaceae plants and affects the colonization of these plants [2,3]. *S. bovinus* has a small-to-medium basidiomata, an orange-brown pileus, a light yellow context that does not change when wounded, and a light yellow subcylindrical stipe (Figure 1). The fruiting body of *S. bovinus* is valued as a tasty food and contains rich protein, vitamins, and a variety of essential amino acids and other nutrients. It also contains anticancer substances, with antitumor, antioxidant, and anti-inflammatory effects, thus providing both medicinal and edible benefits [4,5]. However, with unique growth and development characteristics, the domestication and artificial cultivation of this fungus remain challenging [6,7]. Thus, there is growing interest in understanding the mycorrhizal formation process of *S. bovinus*, as well as the underlying molecular mechanisms to facilitate strategies for the effective cultivation of this ECM fungus.

High-quality reference genomes can serve as crucial resources for mushroom breeding, population genetics, and comparative genomic studies [8]. The analysis of genomic data can provide insight into the genetic characteristics of *S. bovinus* and the genetic mechanisms of the symbiosis with the host, enabling researchers to develop improved strategies for the conservation, reproduction, and sustainable utilization of the fungus. Lofgren et al. [9] identified dynamic genome evolution in host specialist ectomycorrhizal fungi by comparing genomes of *Suillus* species, including *S. bovinus*, but the details of its genome were not determined, and annotation analysis was not performed. Thus, to further study the genome of *S. bovinus*, we conducted whole-genome sequencing and assembled it to the chromosome level.

The main objectives of this study were as follows: (1) to construct a high-quality reference genome of *S. bovinus* and describe its basic characteristics in detail, which lays a foundation for the subsequent functional genome research; (2) to understand the genetic composition and potential functions of genes of *S. bovinus* through annotation analysis using different functional databases (KOG, GO, and KEGG); (3) to clarify the evolutionary relationships of *S. bovinus* and its genetic basis using the phylogenetic analysis of *S. bovinus* and 14 other fungal species and the comparative genomic analysis of important functional genes; (4) to explore the functional genes in the *S. bovinus* genome involved in symbiosis and obtain insight into the mechanism of ECM mycorrhiza formation.

Here, we report a high-quality genome of *S. bovinus* at the chromosome level by combining PacBio HiFi sequencing and Illumina sequencing with Hi-C assisted assembly. This assembly provides important theoretical information for the biological study of edible mushrooms and also provides guidance to explore the mechanism of the symbiosis of ECM fungi. The results of this work should help to further develop the economic and ecological values of *S. bovinus*.

## 2. Materials and Methods

### 2.1. S. bovinus Strains and DNA Preparation

*S. bovinus* SB11 was isolated from a fresh fruiting body collected in Yunnan province, China, and maintained at the Research Institute of Tropical Forestry, Chinese Academy of Forestry. Pure cultures of the strains were grown on a modified Melin–Norkrans (MMN) agar medium (glucose, 10 g; malt extract, 5.0 g; vitamin B1, 0.1 mg; CaCl•2H_2_O, 0.05 g; NaCl, 0.025 g; (NH_4_)_2_HPO_4_, 0.25 g; MgSO_4_•7H_2_O, 0.15 g; KH_2_PO_4_, 0.5 g; FeCl_3_ (1.0% solution), 1.2 mL; agar, 15 g; distilled water to 1000 mL; pH 6.0) at 24 °C in darkness.

For high-quality genomic DNA extraction from strain SB11, vegetative mycelia of *S. bovinus* were cultured in a liquid MMN medium for 14 days at 24 °C, with stirring at 150 rpm in darkness. The mycelia were collected through centrifugation, washed with 0.1 M PBS, and frozen in liquid nitrogen before DNA isolation and sequencing. The genomic DNA of *S. bovinus* was extracted using the cetyltrimethylammonium bromide (CTAB) method [10] and quantified with a Qubit^®^ 2.0 Fluorometer (Thermo Scientific, Carlsbad, CA, USA), and DNA quality was assessed by agarose gel electrophoresis.

### 2.2. Library Construction, Sequencing, and Assembly

After confirmation by electrophoresis, DNA samples were fragmented to the required size using Covaris g-TUBE (Covaris, Inc., Woburn, MA, USA) to construct PacBio-SMRT-Bell, Illumina short reads, and Hi-C libraries. The short reads from the Illumina and Hi-C libraries were controlled for quality [11] and then sequenced on the Illumina NovaSeq-6000 platform (Illumina Inc., San Diego, CA, USA). The SMRT-Bell library was constructed for long-read sequencing, and sequencing was performed on the PacBio Sequel Ⅱ platform (Pacific Biosciences of California, Menlo Park, CA, USA) using the circular consensus sequencing (CCS) model with the parameter “min-passes = 3, min-rq = 0.99”. The output bam file was converted to fastq format, generating 8.16 Gb PacBio HiFi reads. Using the effective HiFi reads of SB11 after quality control, Hifiasm software (https://github.com/chhylp123/hifiasm, accessed on 6 January 2024) was used to carry out genome assembly.

Approximately 5088 Mb of Hi-C clean reads were generated from the Hi-C library and mapped to the SB11 preliminary assembly using Juicer v1.6.2 with default parameters [12]. The data were filtered using a Perl script from LACHESIS (https://github.com/shendurelab/LACHESIS/, accessed on 6 January 2024) [13]. The uniquely mapped data were retained and then linked into pseudochromosomes using ALLHiC software (https://github.com/tangerzhang/ALLHiC, accessed on 6 January 2024) to achieve the chromosome-level assembly of *S. bovinus* [14]. We applied 3D-DNA v180922 to order and orient the clustered contigs [15]. Then, Juicer was used to filter and cluster the sequences, and Juicebox v1.11.08 [12] was applied to manually adjust chromosome construction (Figure S1). Finally, we assessed genome completeness using BUSCO v4.1.2 [16].

### 2.3. Genome Component Prediction

Genome component prediction was performed to predict coding genes, repetitive sequences, and noncoding RNA.

We used the GeneMarkS (http://topaz.gatech.edu/, accessed on 6 January 2024) [17] program to retrieve the related coding genes. Protein-coding sequence prediction was performed using a combination of de novo prediction and similarity to homologous proteins in other *S. bovinus* genomes. Augustus v3.3.3 [18] was used for the de novo prediction of protein-coding genes, and *S. bovinus* genomic information was used for the homology prediction of protein-coding genes. We used RepeatMask software v4.1.0 [19] to predict interspersed repeat (IR) sequences and TRF (Tandem Repeats Finder, v4.07b) [20] to identify tandem repeat (TR) sequences in DNA sequences. Based on the characteristics of noncoding RNA, tRNA was predicted by tRNAscan-SE v2.0.7 [21], and rRNA was predicted by rRNAmmer v 1.2 [22]. Similar prediction principles were used for sRNA, snRNA, and miRNA. The sequences of ribosomal RNAs and other ncRNA were identified with Rfam v9.1 software based on the Rfam database, and then its cmsearch program (default parameters) was used to determine the final sRNA, snRNA, and miRNA [23,24].

### 2.4. Genome Annotation

We used several databases to predict gene functions: Non-Redundant Protein Database (NR) [25], Kyoto Encyclopedia of Genes and Genomes (KEGG) [26], Gene Ontology (GO) [27], Clusters of Orthologous Groups (COG) [28,29], and Swiss-Prot (http://www.ebi.ac.uk/uniprot/, accessed on 6 January 2024) [30]. A whole-genome BLAST search (E-value ≤ 1 × 10^−5^) was performed against the above databases. For the comparison results of each sequence, the comparison result with the highest score was selected (default identity ≥ 40%, coverage ≥ 40%), and that result was annotated to obtain the relevant biological function annotation information.

### 2.5. Gene Family and Phylogenetic Analyses

Gene families of 14 other fungi and *S. bovinus* were constructed using several software programs. All genes were first aligned pairwise with BLAST v2.2.26, and then redundancy was removed with Solar v0.9.6. Then, based on the alignment results, Hcluster sg v0.5.0 [31] was used to cluster gene families. Core and specific genes were analyzed using Cluster Database at High Identity with Tolerance (CD-HIT) v4.8.1 software for the rapid clustering of similar proteins, with a threshold of 50% pairwise identity and 0.7 length difference cutoff in amino acid [32].

A total of eight ectomycorrhizal mushrooms and six saprophytic mushrooms were selected to construct a genome-wide phylogenetic tree (Table S1). Core-pan analysis was used to identify the single copy core gene of the samples, and MUSCLE v5.1 software was used to compare the protein multiple sequences and convert them into CDS. Next, a phylogenetic tree was constructed using Treebest v1.9.2 software using the maximum likelihood method, with a bootstrap setting of 1000 with homologous genes [33]. The phylogenetic tree was constructed using Mega v6.06 for drawing adjustments and editing [34]. *Tuber melanosporum* was an outgroup in the evolutionary tree.

The target and reference genomes were compared using MUMmer v3.23 [35] software, and large-scale covariance relationships between genomes were identified. Next, an inter-region comparison was performed using LASTZ v1.03.54 [31,36] to confirm the local positional alignment relationships.

### 2.6. Comparative Analysis of Carbohydrate-Active Enzymes

The *S. bovinus* genome sequence was analyzed to determine the presence of carbohydrate-active enzymes (CAZymes). Genes encoding CAZymes [37] in the *S. bovinus* genome were annotated using the Carbohydrate-Active Enzymes (CAZy) database and the dbCAN2 annotation tool (http://bcb.unl.edu/dbCAN2/, accessed on 6 January 2024) [38] through the HMMER method, with parameters set as E-value < 1 × 10^−15^ and coverage > 0.35.

### 2.7. Secondary Metabolite Gene Clusters in S. bovinus

Secondary metabolite gene clusters (SMs) were identified using AntiSMASH fungal 7.1.0 (https://fungismash.secondarymetabolites.org/#!/start/, accessed on 6 January 2024) [39], a web-based analysis platform, using the default parameter settings.

### 2.8. Secreted Protein Predictive Analysis

The secreted proteins (SPs) were predicted using SignalP v5.0 [40] and TMHMM v2.0c [41] tools to identify signal peptides and transmembrane structures. Screening for secretory proteins with less than 300 amino acids as small secretory proteins (SSPs) was conducted [9].

## 3. Results and Discussion

### 3.1. Subsection Genome Sequencing and Assembly

The genome of *S. bovinus* was sequenced using the Illumina NovaSeq 6000 (Illumina Inc., San Diego, CA, USA) and PacBio Sequel II platforms(Pacific Biosciences of California, Menlo Park, CA, USA). Approximately 8.16 Gb of PacBio HiFi raw data were obtained. Illumina and Hi-C libraries were sequenced on the Illumina NovaSeq-6000 platform, producing 2.15 Gb and 5.09 Gb of raw data, respectively (Table S2). Genome assembly was performed using Hifiasm software (https://github.com/chhylp123/hifiasm, accessed on 6 January 2024), and then the ALLHiC software(https://github.com/tangerzhang/ALLHiC, accessed on 6 January 2024) was employed for genome sequence clustering, sorting, and orientation with the application of the HTC-hicker contig direction correction. In the final assembly, the *S. bovinus* genome was organized into 12 chromosomes, with a total length of 43.03 Mb and a contig N50 size of 3.78 Mb.

The chromosome-anchoring rate of contigs longer than 100 bp was 93.92% (Table 1; Figure S1). The genome integrity of *S. bovinus* was evaluated using BUSCO 3.0.2. Of the 290 BUSCO groups, 278 complete BUSCOs (94.5%) were identified, including 274 complete single-copy BUSCOs and 4 complete duplicate BUSCOs (Table S3). These results indicated that the *S. bovinus* genome assembly had relatively high continuity and integrity, sufficient for the next steps of analysis and annotation. Overall, the genomic metrics and mount rates were favorable.

**Figure 2:**
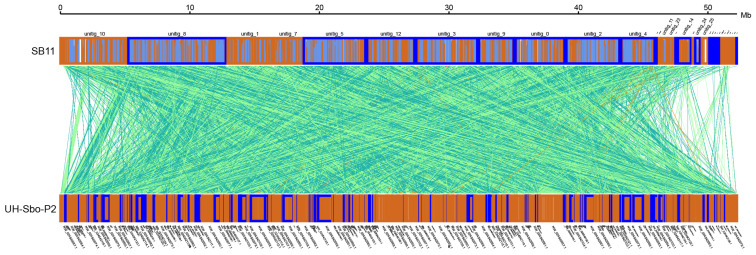
Synteny of *S. bovinus* SB11 with *S. bovinus* UH-Sbo-P2. The forward and reverse strands are represented by orange boxes and blue boxes, respectively.

A comparison of the *S. bovinus* SB11 strain with the sequenced *S. bovinus* UH-Sbo-P2 strain showed good continuity of the *S. bovinus* SB11 genome in N50 length, much better than the N50 length of 0.32 Mb of the *S. bovinus* UH-Sbo-P2 strain. However, the SB11 genome was smaller than the UH-Sbo-P2 genome. The genomic synteny analysis of SB11 and UH-Sbo-P2 strains was performed using MUMmer v3.23 and LASTZ v1.03.54 software. The results showed that SB11 and UH-Sbo-P2 had a large number of homologous genes with high collinearity (73.05%), but gene rearrangements such as gene loss and translocation were also detected (Figure 2). This may be due to partial gene loss in the SB11 strain under the selective pressure of different environments such as host, climate, or geographical location, or may reflect differences in the number of repeat sequences, leading to differences in genome size. In addition, differences in sequence data might arise from the use of different sequencing instruments and sequencing methods, so the basis of identified differences should be further analyzed.

### 3.2. Genome Information at Chromosome Level

The analysis of the HiC data was performed to identify contigs sequences and these sequences were mounted to the chromosome level using ALLHiC software (https://github.com/tangerzhang/ALLHiC, accessed on 6 January 2024). The 12 chromosomes of the assembled *S. bovinus* genome were further analyzed, and the detailed sequence distribution of each chromosome is shown in Table 2. The results showed that the 12 chromosomes were of different sizes, with an average length of 3,585,466 bp. Chromosome 9 had the longest sequence length of 4,909,462 bp, followed by chromosome 11 with 4,752,148 bp, and chromosome 5 had the shortest length of 2,235,747 bp. In total, 11,999 coding genes were annotated. Chromosome 9 had the most coding genes with 1399 genes, followed by chromosome 10 with 1345 genes, and chromosome 5 had the fewest genes with 550 genes.

### 3.3. Genomic Component Analysis

The genome was annotated using a combination of de novo and homology-based approaches. A total of 11,199 coding genes were obtained through an integrative prediction of coding genes, with an average coding gene length of 1631 bp (Table 1). Based on the assembled genome sequence and the prediction of coding genes, Circos v0.69-9 software was used to construct a circular genome diagram of *S. bovinus*. Meanwhile, the noncoding RNA and gene prediction were analyzed, and the results are shown in Figure S2.

#### 3.3.1. Repeat Sequence Annotation

The repeat sequences of *S. bovinus* SB11 were subdivided into interspersed repeats (IRs) and tandem repeats (TRs) (Table 3). A total of 4056 IRs, 1,242,627 bp in length, were predicted, accounting for 2.8881% of the SB11 genome length. The scattered repeats included short interspersed nuclear elements (SINEs), long interspersed nuclear elements (LINEs), long terminal repeats (LTRs), DNA transposons, and rolling circles (RCs), accounting for 0.0033% (18), 0.1062% (470), 2.5465% (2796), 1.707% (625), and 0.0629% (129), respectively. TRs represented 1.7932% of the assembled genome. The microsatellite DNA and minisatellite DNA accounted for 0.0280% and 0.4563% of the assembled genome, respectively, in the TRs.

#### 3.3.2. Annotation of Noncoding RNA Genes

Noncoding RNAs (ncRNAs) perform a variety of biological functions. These RNAs are not translated into proteins but function directly at the RNA level. For microorganisms, the most commonly studied ncRNAs are sRNA, rRNA, and tRNA. Several noncoding RNAs were predicted in the *S. bovinus* genome, as shown in Table 4. There were 138 tRNAs and 15 rRNAs predicted. Among the rRNA sequences, there were five 5s_rRNAs, five 18s_rRNAs, and five 28s_rRNA.

### 3.4. Gene Function Annotation

The predicted protein sequences of 11,199 coding genes were compared, and annotated using data from six public databases (GO, KEGG, KOG, NR, Pfam, and Swiss-Prot). The Nr database was used to match the most genes (10,793 genes, or 96.37%), followed by those from KEGG (7290 genes, or 65.10%), GO (6545 genes, or 58.44%), Pfam (6545 genes, or 58.44%), Swiss-Prot (2140 genes, or 19.11%), and KOG (1640 genes, or 14.64%) (Table S4).

#### 3.4.1. KOG Annotations

**Figure 3:**
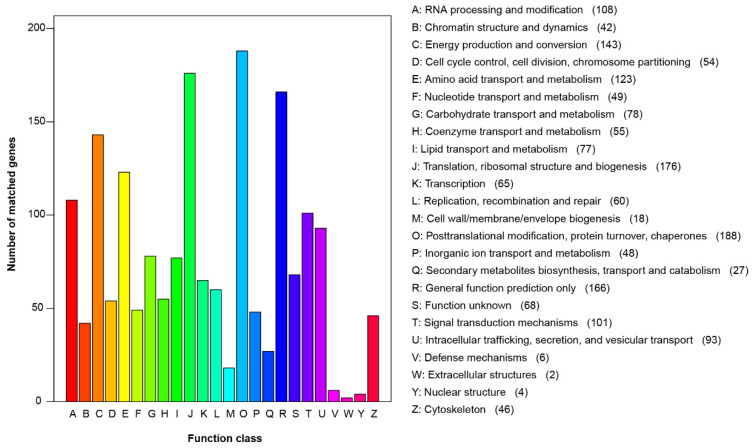
The KOG functional annotation of *S. bovinus*.

The analysis of the *S. bovinus* SB11 genome with the KOG database allowed for the annotation of 1640 genes and classification into 24 categories (Figure 3). The top five were “Posttranslational modification, protein turnover, chaperones” (188, 11.46%), “translation, ribosomal structure and biogenesis” (176, 10.73%), “general function prediction only” (166, 10.12%), “energy production and conversion” (143, 8.72%), and “amino acid transport and metabolism” (123, 7.5%). These findings suggest rich and diverse protein and energy metabolic functions in *S. bovinus*, allowing for the effective absorption and transformation of nutrients in the substrate. In addition, the analysis revealed 68 genes with “unknown functions” that require further study.

#### 3.4.2. GO Annotations

**Figure 4:**
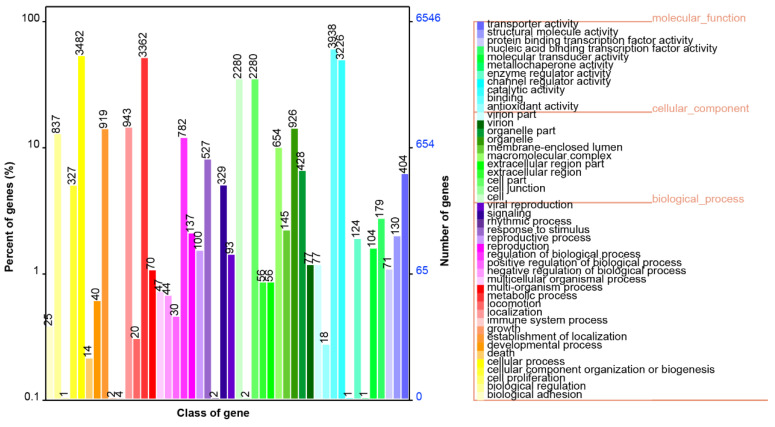
GO function annotation of *S. bovinus*.

In the *S. bovinus* genome, a total of 6545 genes were annotated to the GO database, distributed among the three functional categories of molecular function, cellular components, and biological processes. In biological processes, 3482 genes participate in the “cell process”, followed by the “metabolic process” (3362), “localization” (943), and the “regulation of biological processes” (782). In the function of cell components, 2280, 2280, 926, and 654 genes were associated with “cell”, “cell part”, “organelle” and “macromolecular complex”, respectively. Among the genes related to molecular function, 3938, 3226, 404, and 179 genes participated in “binding”, “catalytic activity”, “transport activity”, and “nucleic acid binding transcription factor activity”, respectively (Figure 4). In the GO functional annotation results, more genes were associated with molecular functions and related to biological processes. The antioxidant capacity of edible macro-fungi has been receiving more attention as a natural resource of bioactive compounds. It is worth noting that, in the *S. bovinus* genome, we found 18 genes involved in “antioxidant activity”. This suggested that *S. bovinus* may have some antioxidant capacity, which in turn regulates the production of some antioxidant substances.

#### 3.4.3. KEGG Annotations

**Figure 5:**
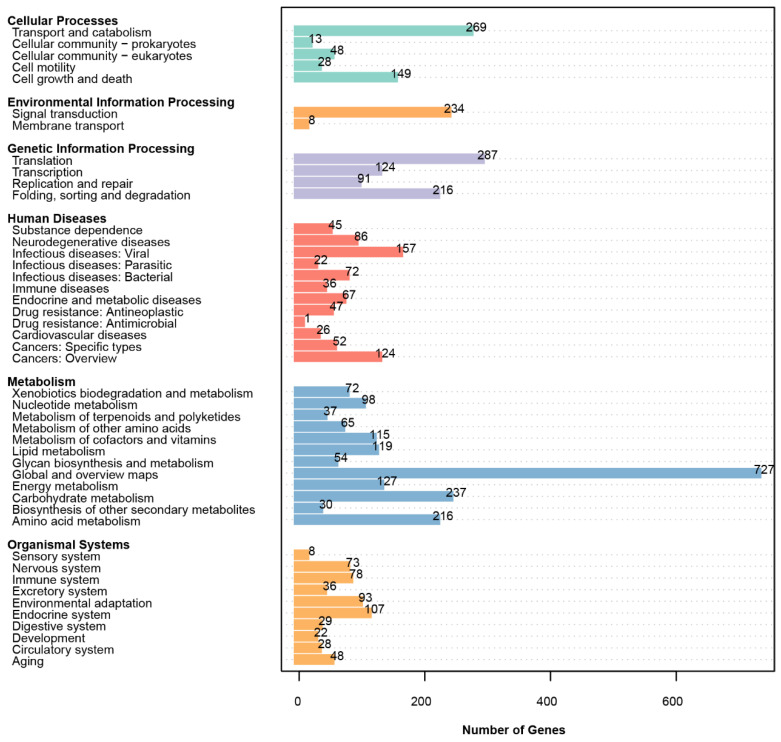
KEGG pathway annotation of *S. bovinus*.

To further analyze the metabolic pathways and functions of *S. bovinus* gene products and compounds in cells, the KEGG database was used. A total of 7290 genes were annotated in the KEGG database, with distribution in six major metabolic pathways of metabolism (12 branches, 1897), genetic information processing (4 branches, 718), environmental information processing (2 branches, 242), cellular processes (5 branches, 507), human diseases (12 branches, 735), and organic systems (10 branches, 522). The number and function of genes in each category are shown in Figure 5. These results suggested the presence of many metabolic genes in *S. bovinus*, with a wide variety of metabolic functions and the potential to act in multiple material metabolic pathways.

### 3.5. Gene Family and Phylogenetic Analysis

**Figure 6:**
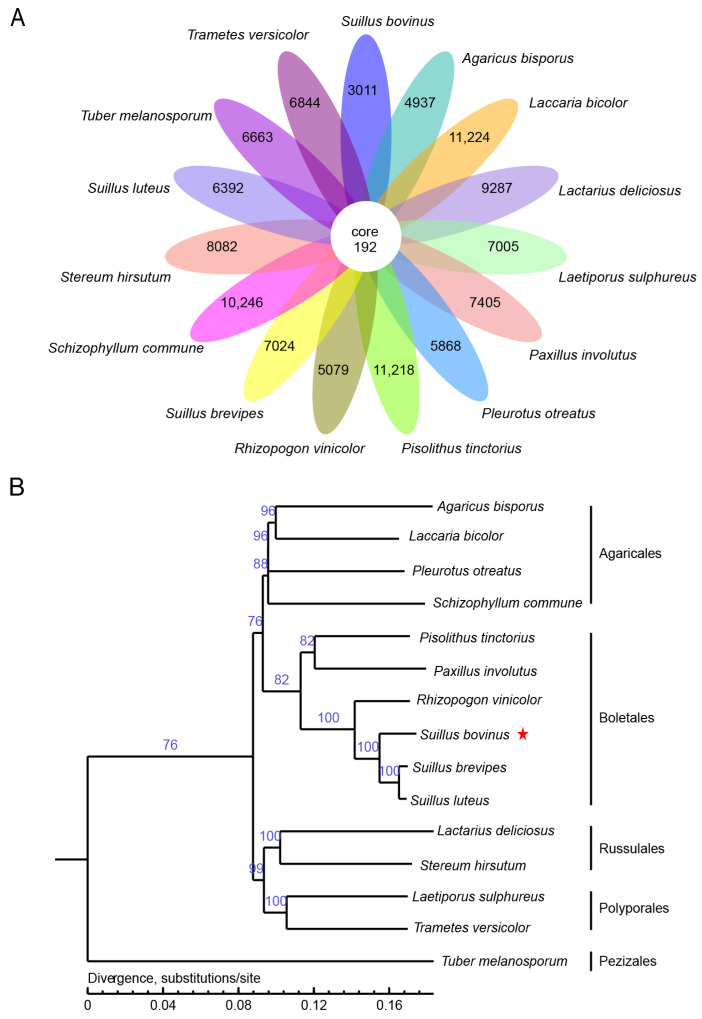
Comparative genomics analysis: (**A**) conserved and specific gene counts (each ellipse represents a strain, and the numbers in the ellipses are the specific genes for that strain. The central white circle represents conserved genes among the 15 strains); (**B**) maximum likelihood phylogenetic tree. The stars are labeled as the target species sequenced in this paper.

Gene family comparisons were performed to obtain insight into the mechanisms of mycorrhizal symbiosis and the nutritional composition of mycorrhizal edibles. The genome of *S. bovinus* and the genomes of 14 other edible fungi were selected for gene family analysis. Using the whole-genome protein-coding gene sequence, the gene families were identified by sequence clustering analysis, and the homologous genes of the 15 fungi were compared to find the core genes, which should have important and conserved biological functions. A total of 128,566 genes were identified, with 192 genes common to all fungi and 3011 protein clusters specific to *S. bovinus* (Figure 6A). These genes may be related to the environmental adaptation and the synthesis of metabolites in *S. bovinus.*

To further study the phylogenetic relationships of *S. bovinus*, a genome-wide phylogenetic tree was constructed based on the homologous genes in the 14 other fungi described above. The phylogenetic tree was constructed with Treebest v1.9.2 software using the maximum likelihood method, with a bootstrap setting of 1000 with homologous genes [33]. *T. melanosporum* was used as an outgroup of the evolutionary tree. The results showed that *S. bovinus* was clustered into a clade with other species of Boletales, with *S. brevipes* and *S. luteus* as its closest relatives (Figure 6B). Although relatively few species were used to generate this phylogenetic tree, the evolutionary relationship is consistent with that of phylogenetic trees constructed based on large-scale species [42].

### 3.6. Carbohydrate Active Enzymes

**Figure 7:**
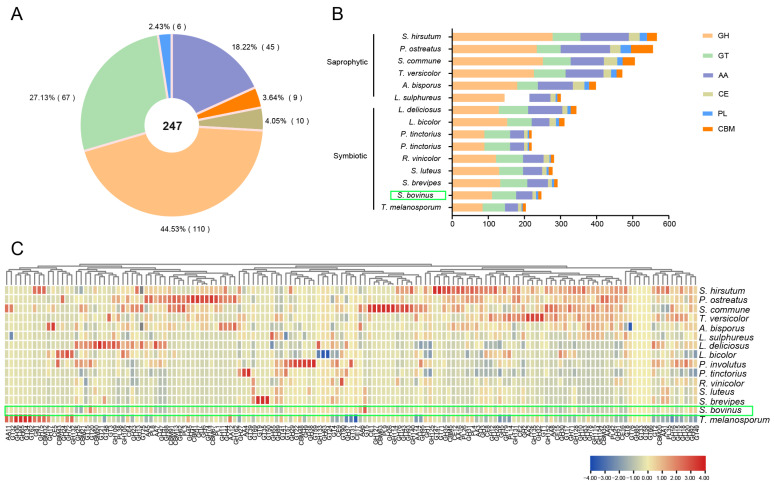
CAZymes in *S. bovinus* and other 14 fungi: (**A**) the distribution of CAZyme categories in *S. bovinus*; (**B**) the distribution of CAZymes in other 14 fungi; (**C**) gene count heatmap of CAZyme subfamilies; the color scale represents the count of the gene normalized by the Z-score method. GH, glycoside hydrolase; GT, glycosyltransferase; PL, polysaccharide lyase; CE, carbohydrate esterase; CBM, carbohydrate-binding module; AA, auxiliary activity. The green boxes are the target species sequenced in this paper.

Carbohydrate-active enzymes (CAZymes) are an important fungal gene family in the fungal genome. CAZymes degrade carbohydrates, lignin, and hemicellulocellulose, allowing fungi to absorb carbon source nutrients and adapt to the environment [43,44,45]. In general, there are five main categories of CAZymes: glycoside hydrolases (GHs), glycosyltransferases (GTs), polysaccharide lyases (PLs), carbohydrate esterases (CEs), and auxiliary activity proteins (AAs). This group also contains carbohydrate-binding modules (CBMs). There is significant interest in the formation mechanism and host selection of ectomycorrhiza. Comparative genomics has revealed differences in CAZymes in the genomes of ectomycorrhizal fungi [10]. Using the annotation of the CAZy database and the dbCAN tool ( http://bcb.unl.edu/dbCAN2/, accessed on 6 January 2024), 247 genes were annotated as CAZymes of *S. bovinus*, accounting for 2.22% of the total genes in the genome (Figure 7A). These CAZyme genes belong to 85 subfamilies, including 110 GHs, 67 GTs, 45 AAs, 10 CE, 9 CBMs, and 6 PLs genes. These results indicated that the content of GHs was the highest, followed by GTs, and the content of PLs was the least in the *S. bovinus* genome (Figure 7A).

We next comprehensively compared the distribution and quantity of CAZyme families of *S. bovinus* with that of eight other ectomycorrhizal fungi and six saprophytic fungi, especially those that degrade cellulose, hemicellulose, and lignin. There were dramatically fewer genes in the CAZyme family of mycorrhizal fungi compared with that of saprophytic fungi, with fewer GHs and AAs than those of saprophytic fungi (Figure 7B). In particular, there were significantly fewer AA2, AA9, GH3, GH43, and CE16 in symbiotic fungi than in saprophytic fungi (Table S5). These enzymes are mainly involved in the degradation of lignin, cellulose, hemicellulose, and pectin, so they are important in the degradation of plant cell wall components [46,47]. A limited number of CAZymes may correspond to less damage to plant cell walls, which is conducive to the establishment of symbiotic relationships between mycorrhizal fungi and host plants [42,48]. Therefore, the absence of these genes may be consistent with a symbiotic nutritional lifestyle.

We further analyzed these protein families by clustering (Figure 7C), and proteins with similar functions clustered together. There were significantly more CAZYs in saprophytic fungi than in symbiotic fungi, mainly concentrated in GHs, namely GH45, CBM1, GH7, GH6, GH74, CBM67, and PL1 clusters, and most of these were in saprophytic fungi, with few in symbiotic fungi (Figure 7C). These proteins are mainly involved in the degradation of cellulase, hemicellulase, and pectinase. In addition, the annotation results showed that GH36, GH65, GT34, and GT62 were only found in *T. melanosporum* but not in other species, which might be because *T. melanosporum* is Ascomycetes, and the other species are Basidiomycetes (Table S5).

Very few genes encoding GH7, GH53, GH115, GH154, CE1, and CBM35 were found in the ectomycorrhizal fungi, but these genes were present in saprophytic fungi. GH6, GH11, GH74, CE15, CBM1, and PL42 were mostly present in saprophytic fungi and missing only in individual species but were largely absent in symbiotic fungi (Table S5). Previous genomic analyses have revealed that multiple ectomycorrhizal fungal lineages lost most of the genes encoding lignocellulose-degrading enzymes present in their saprotrophic ancestors [42,48,49,50]. The loss of these genes might be associated with the evolution of symbiotic fungi, creating a more favorable environment for the establishment of mutualistic relationships between fungi and host plants. Although the *S. bovinus* genome lacks the enzymes involved in degrading the plant cell wall, such as the GH6 and GH7 families, the GH5 family is present. This is similar to what is found in ectomycorrhizal fungi such as *Laccaria bicolor* and *T. melanosporum* (Figure 7C and Table S5) [51,52]. Previous studies showed that the *T. melanosporum* genome retained the cellulolytic enzyme gene TmelCMC3 (GH5) but lacked GH6 and GH7, and the GH5 gene was heavily upregulated during symbiosis with *Corylus avellana*, suggesting that GH5 might be a key enzyme involved in cell wall penetration [53]. These results indicate that S. *bovinus* may have some ability to decompose and utilize lignocellulose as a nutrient source. However, hemicellulose-degrading enzymes (GH10, GH11, GH74, GH93, and GH115) and pectin-degrading enzymes (GH78, GH93, PL1, PL3, and PL4) were not identified in the *S. bovinus* genome. These enzymes play key roles in the degradation of plant cell wall components [46,47]. Thus, our analysis revealed that *S. bovinus* lost most of the genes that degrade plant cell wall polysaccharides (cellulose, hemicellulose, and pectin), and this may be related to its adaptation to a symbiotic lifestyle.

When mycorrhizal fungi are in a symbiotic relationship with a host plant, most of the carbohydrates required for mycorrhizal fungi growth come from the glucose provided by the host plant, so the fungi do not need to degrade lignocellulose in the environment to obtain nutrients [54]. Therefore, we investigated CAZymes encoding α-amylase (e.g., GH13, GH70, GH77, GH57, and GH119 families) and sucrose invertase (GH32) in *S. bovinus*. GH70, GH77, GH57, and GH119 were not found in the *S. bovinus* genome, but nine GH13 genes were identified, suggesting that *S. bovinus* may be able to degrade starch. We also found that *S. bovinus*, like most ectomycorrhizal fungi, lacks the sucrose invertase GH32 gene. This means that *S. bovinus* is unable to utilize sucrose directly from the plant, so it is completely dependent on its partner for glucose [42].

### 3.7. Secondary Metabolisms

Fungi can produce abundant secondary metabolites, such as terpenes, steroids, steroids, phenolic acids, and fatty acids [55]. Secondary metabolites in fungi are mainly regulated by polyketide synthase (PKS), nonribosomal peptide synthetase (NRPS), hydrogenase, oxidase, and transporters [56]. Terpenoids such as sesquiterpenes, diterpenes, triterpenes, and terpenes act in the recognition and reaction between fungi and plants and are recognized as key compounds in ectomycorrhiza, especially sesquiterpenoids [55,57].

Auxin and lipochitooligosaccharides may function as signaling molecules for symbiosis between ECM fungi and host plants [58,59,60,61]. In symbiosis with plants, ECM fungi can secrete auxin into plant root cells to inhibit the growth of the primary root of the host plant and induce the production of lateral roots [60]. ECM fungi can also be recognized and activated by host plants through secreted lipochitooligosaccharides and chitooligosaccharides, which are found in ECM fungi, rhizobia, and arbuscular mycorrhizal fungi [49,61,62].

#### 3.7.1. Terpene Biosynthesis

The webtool antiSMASH v7.1.0 was used to predict the secondary metabolites of the *S. bovinus* genome and 14 other fungi. Our results showed that *S. bovinus* has 31 gene clusters potentially involved in the biosynthesis of secondary metabolites. This is higher than most symbiotic fungi (average 20.89) and saprophytic fungi (average 27.50). However, the diversity of gene clusters in *S. bovinus* was lower than that of other fungi. The 31 gene clusters included only three types, with 16 terpenoid gene clusters, 12 NRPS-like gene clusters, and 3 T1pks gene clusters (Table 5). This is consistent with the results of Lofgren et al. [9] indicating that *Suillus* has a higher number of SM gene clusters and less SM gene cluster diversity than other ECM fungi.

Previous studies [58,61] showed that ECM fungi can release sesquiterpenes and terpene derivatives to promote the formation of lateral roots in the host plant, e.g., the release of sesquiterpenes by the *Laccaria bicolor* promotes the formation of poplar lateral roots. A total of 16 gene clusters related to terpenoid biosynthesis were detected in the *S. bovinus* genome. We detected 19 *S. bovinus* genes in the “terpenoid backbone biosynthesis (map00900)” pathway (Table 5). The MVA and MEP/DOXP pathways make up the main part of map00900 (Figure S3). These results suggest that terpenoid backbone biosynthesis in *S. bovinus*, like in most fungi, can only proceed through the MVA pathway [50,63,64]. The core enzymes involved in the MVA pathway are listed in Table 6.

We also detected the “sesquiterpenoid and triterpenoid biosynthesis (map00909)” pathway in *S. bovinus*, with five genes in this pathway (Table 6; Figure S4). This indicates that *S. bovinus* may be similar to *L. bicolor*, which secretes sesquiterpenoids to stimulate the growth of lateral roots of the host plant during symbiosis. These compounds might act as symbiotic signals with the host plant [58]. Eleven genes were identified in the “ubiquinone and other terpenoid quinone biosynthesis (map00130)” pathway (Table 6; Figure S5), indicating that *S. bovinus* might be able to synthesize ubiquinone [65,66].

**Figure 8:**
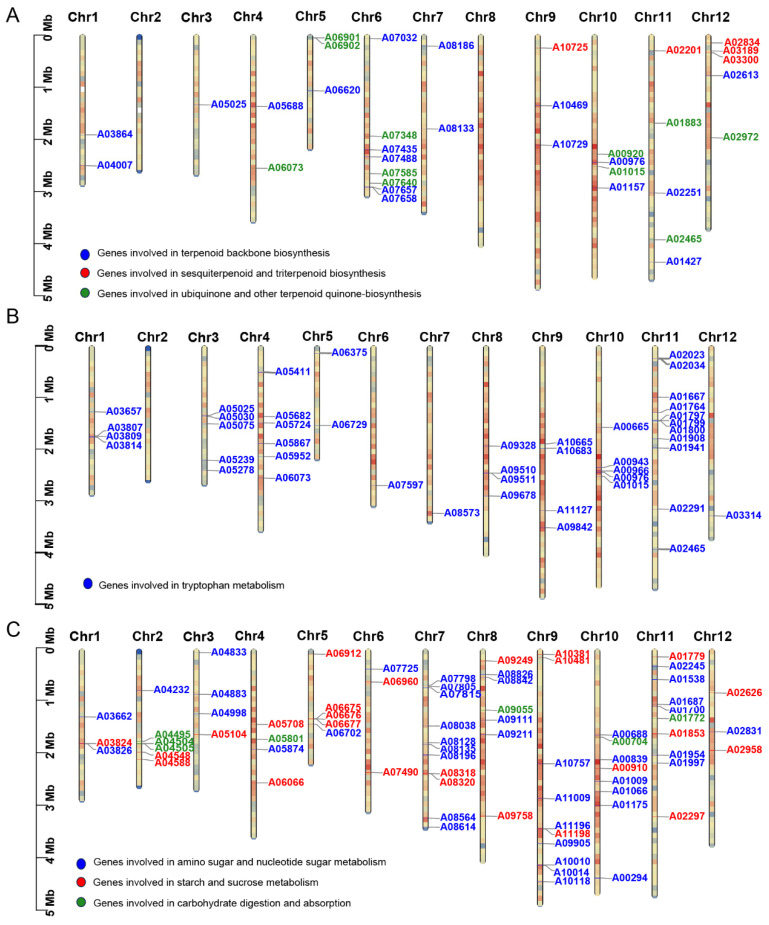
Distribution of *S. bovinus* symbiosis-related genes on chromosomes: (**A**) the putative terpene synthase genes. Blue represents genes involved in terpenoid backbone biosynthesis (map00900); red represents genes involved in sesquiterpenoid and triterpenoid biosynthesis (map00909); green represents genes involved in ubiquinone and other terpenoid quinone biosynthesis (map00130); (**B**) the putative genes involved in tryptophan metabolism (map00380); (**C**) the putative genes involved in lipochitooligosaccharide biosynthesis. Blue represents genes involved in amino sugar and nucleotide sugar metabolism (map00520); red represents genes involved in starch and sucrose metabolism, (map00500); green represents genes involved in carbohydrate digestion and absorption (map04973).

In order to determine the positions of the above genes related to terpene synthesis on chromosomes, we used tbtools v1.9.2 software to locate the above genes. The results showed that these genes were unevenly distributed on 10 chromosomes and clustered together in tandem, and there were no terpene-related genes on Chr2 and Chr8 (Figure 8A). Among them, the terpenoid backbone pathway genes (19 genes) were located on the 10 chromosomes. The sesquiterpene and triterpene genes (five genes) were mainly distributed in the upper part of Chr9 (one gene), Chr11 (one gene), and Chr12 (three genes). Other terpenoid and ubiquinone genes were mainly distributed on Chr5, Chr6, and Chr10, with one gene each on Chr4, Chr11, and Chr12.

#### 3.7.2. Auxin Metabolism

Auxin synthesis is largely dependent on the tryptophan pathway [67]. In the *S. bovinus* genome, we identified 44 genes related to tryptophan metabolism (map00380) and associated with auxin metabolism (Figure 8B). The genes involved in the tryptophan metabolism pathway were unevenly distributed on 11 chromosomes, with no tryptophan metabolism-related genes on Chr2. ECM fungi such as *L. bicolor* and *T. melanosporum* can produce a large amount of auxin IAA, thus causing morphological changes in symbiotic plant roots [61]. This change was due to direct contact between ECM fungi and plant roots or indirect diffusion signals from the fungi. *L. bicolor* can secrete auxin into root cells, inhibiting the growth of the host taproot and inducing the formation of lateral roots of poplar [60]. IAA was detected in the fermentation broth of *S. bovinus*, with significantly higher IAA in the host plant than in the control plant during the symbiosis process [68]. These results suggest that *S. bovinus* is similar to other ectomycorrhizal fungi and can synthesize and secrete auxin to achieve symbiosis with host plants.

#### 3.7.3. Lipochitooligosaccharide Biosynthesis

In the *S. bovinus* genome, we found 29, 48, and 7 genes involved in starch and sucrose metabolism, (ko00500), amino sugar and nucleotide sugar metabolism (ko00520), and carbohydrate digestion and absorption (ko04973), respectively, and these genes were unevenly distributed across the 12 chromosomes (Figure 8C). Among them, the genes related to amino sugar and nucleotide sugar metabolism (map00520) were distributed on all 12 chromosomes, with most distributed on Chr7, 9, 10, and 11. The genes related to starch and sucrose metabolism (map00500) were unevenly distributed on 12 chromosomes, and the highest distribution was on Chr5 with four related genes. The genes related to carbohydrate digestion and absorption (map04973) were only distributed on Chr2, 4, 8, 10, and 11, with the most on Chr2 and only one gene on each of chromosomes 4, 8, 10, and 11.

These metabolic processes are closely related to the biosynthesis of lipochitooligosaccharides and may be involved in signaling as part of the symbiotic system formation of *S. bovinus*. Lipid chitooligosaccharides secreted by the ECM fungus L. bicolor can partially activate the generalized symbiotic signaling pathway, affecting the formation of lateral roots of plants [61,69], and these metabolites might play similar roles in *S. bovinus*.

### 3.8. Secretory Protein Analysis

Secretory proteins (SPs) are important enzymes required for life activities. These proteins are synthesized inside the cell and then secreted outside the cell across the cell membrane under the guidance of a signal peptide. Secretory proteins smaller than 300 amino acids are known as small secretory proteins (SSPs) [49], and SSPs play key roles in symbiosis in some ECM fungi [70].

**Figure 9:**
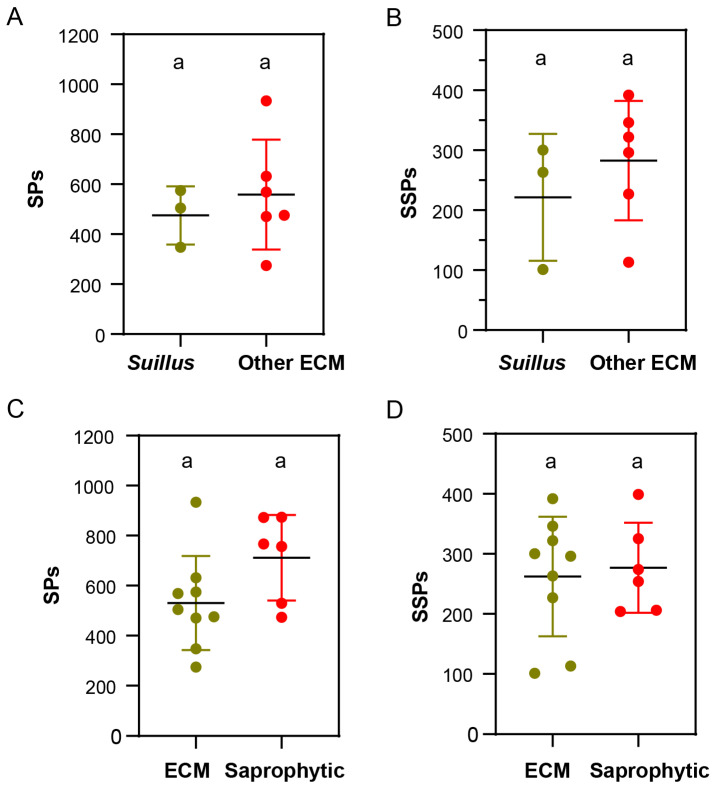
Secreted protein (SP) and small secreted protein (SSP) distributions for *S. bovinus* and 14 other fungi. Note: different letters indicate significant differences between groups: (**A**,**B**) *Suillus* fungi vs. other ECM fungi; (**C**,**D**) ECM fungi vs. Saprophytic fungi.

Differences in the number of SPs and SSPs between *S. bovinus* and 14 other fungi were predicted by using a combination of bioinformatics software SignalP 5 and TMHMM 2.0c. *Suillus* contained fewer SPs and SSPs than other ECM fungi, but the difference was not significant (Figure 9A,B), a finding that was consistent with those of Lofgren et al. [9]. There were also no significant differences in SPs and SSPs between the ECM fungi and saprophytic fungi, though overall, there were fewer SPs in ECM fungi than in saprophytic fungi (Figure 9C,D). Further analysis revealed that *S. bovinus* had fewer secreted proteins than other fungi, with 347 SPs predicted, of which 101 were SSPs.

**Figure 10:**
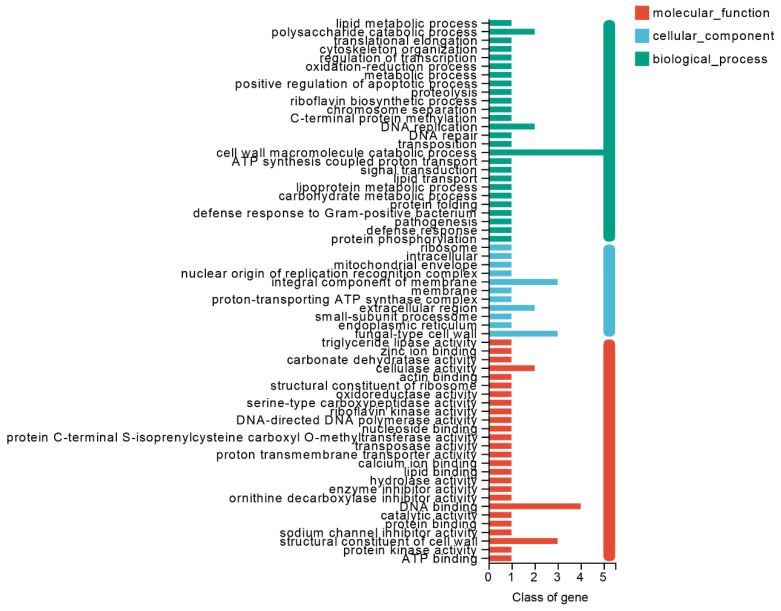
The GO function annotation of small secreted proteins (SSPs) in *S. bovinus*.

The GO annotation analysis of the SSPs of *S. bovinus* yielded a total of 30 annotated SSPs and 64 GO terms, including 27 associated with biological processes, 26 molecular functions, and 11 cellular components (Figure 10). The most abundant biological processes were the “cell wall macromolecule catabolic process” (5), “DNA replication” (2), and “polysaccharide catabolic process” (2). The most frequent molecular functions were “DNA binding” (4), “structural constituent of cell wall” (3), and “cellulase activity” (2). The most frequent terms for cellular component categories were “endoplasmic reticulum” (3), “integral component of membrane” (2), and “extracellular region” (2). These SSPs might participate in *S. bovinus* symbiosis. SSPs are major effectors of pathogenic and symbiotic fungal–host interactions [71], and signaling, nutrient transport, and nutrient metabolism-related genes in ECM fungi can be significantly upregulated during the occurrence of symbiosis [10]. MiSSP7 of *L. bicolor* is required for the establishment of symbiosis, and MiSSP interacts with the JAZ6 to inhibit the plant defense response and promote the establishment and development of ectomycorrhizal roots [71,72]. In addition to MiSSP7, other partially characterized SSPs include LbMiSSP7.6, LbMiSSP8, and PaMiSSP10b. Further identification and characterization of SSPs is crucial for the study of mycorrhizal symbiosis mechanisms [73,74,75]. *S. bovinus*, as an ECM fungus, needs to avoid rejection by the host’s immune response, and signaling substances such as SSPs might be important for escaping detection. Unfortunately, SSPs previously identified as acting in symbiosis were not found in the *S. bovinus* genome. This suggests that *S. bovinus* has its own specific SSPs, the characterization of which will be a focus of future work.

## 4. Conclusions

In summary, this is the first report of a chromosomal-level assembly of *S. bovinus*. Using sequencing and Hi-C data, we accurately anchored ~93.92% of the whole-genome sequences into 12 chromosomes. Integrity and completeness analysis revealed the high quality of this genome assembly. We hope that the genomic information and analysis can provide a reference for the molecular study of the symbiosis mechanism of *S. bovinus* and enable the study of the genetic evolutionary relationships of *S. bovinus*.

The genome of *S. bovinus* enables us to predict gene function and study the biosynthesis of active compounds. The current work revealed the genetic basis for the degradation of lignin, cellulose, and aromatic compounds in *S. bovinus*. The comparative genomic analysis of the gene families, carbohydrate enzymes, and secondary metabolism will help guide future work to investigate the symbiotic mechanism and saprotrophic capacity of *S. bovinus*. It is crucial to annotate the genes involved in terpene, auxin, and lipochitooligosaccharide biosynthesis to understand the mechanisms of valuable secondary metabolite production and the diversity of major components. This work provides high-quality reference genomic resources for resource utilization and research on mycorrhizal symbiosis mechanisms.

## Figures and Tables

**Table 1:** Whole-genome assembly features of *S. bovinus*.

Sample_ID	S. *bovinus* SB11 *	S. *bovinus* UH-Sbo-P2 **
Sequencing technology	PacBio CCS, Illumina, Hi-C	PacBio
Genome size (Mb)	43.03	47.50
Contigs Num	18	622
Contigs N50_Length (bp)	3,780,628	320,421
GC (%)	46.58	46.9
pseudo chromosome	12	-
Number of coding genes	11,199	13,537
chromosome anchoring rate for contigs (%)	93.92	-
Assembly level	Chromosomes	Contig

Note: * this study, ** Lofgren et al., 2021 [9], - not detected.

**Table 2:** Summary of Hi-C-assisted assembly chromosome lengths of *S. bovinus.*

Sequence ID	Sequence Length (bp)	Number of Coding Genes
chromosome1	2,921,455	703
chromosome2	2,669,988	625
chromosome3	2,726,889	626
chromosome4	3,625,200	978
chromosome5	2,235,747	550
chromosome6	3,144,253	821
chromosome7	3,462,440	899
chromosome8	4,096,261	1162
chromosome9	4,909,462	1399
chromosome10	4,701,129	1345
chromosome11	4,752,148	1153
chromosome12	3,780,628	938

**Table 3:** Statistical analysis of repeat sequences in the *S. bovinus* genome.

Repeat Type	Type	Number (#)	Total Length (bp)	Repeat Size (bp)	In Genome (%)
Interspersed repeats	LTR	2796	1,095,626	-	2.5465
	DNA	625	73,438	-	0.1707
	LINE	470	45,708	-	0.1062
	SINE	18	1414	-	0.0033
	RC	129	27,079	-	0.0629
	Unknown	18	1605	-	0.0037
Tandem repeats	TR	4672	563,158	1~592	1.3089
	Minisatellite DNA	2919	196,344	10~60	0.4563
	Microsatellite DNA	208	12,040	2~6	0.0280

Note: -, not detected.

**Table 4:** Statistical analysis of noncoding RNAs in the *S. bovinus* genome.

Type	Number (#)	Average Length (bp)	Total Length (bp)	Percentage of Genome (%)
tRNA	138	81	11,257	0.0262
5s_rRNA	5	114	570	0.0013
5.8s_rRNA	0	0	0	0.0000
18s_rRNA	5	2210	11,051	0.0679
28s_rRNA	5	3520	17,598	0.0409
sRNA	0	0	0	0.0000
snRNA	0	0	0	0.0000
miRNA	0	0	0	0.0000

**Table 5:** Comparison of Secondary metabolisms of *S. bovinus* with those of other fungi.

Secondary Metabolisms	Terpene	NRPS	Siderophore	T1pks	Indole	Fungal-RiPP	Total
*Suillus bovinus*	16	12	0	3	0	0	31
*Laccaria bicolor*	8	4	1	1	1	0	15
*Pisolithus tinctorius*	12	7	0	1	0	1	21
*Suillus brevipes*	3	5	0	1	0	0	9
*Suillus luteus*	12	10	0	2	0	0	24
*Tuber melanosporum*	3	3	0	1	0	0	7
*Paxillus involutus*	7	7	0	3	0	0	17
*Rhizopogon vinicolor*	21	18	0	1	0	1	41
*Lactarius deliciosus*	18	2	2	1	0	0	23
*Trametes versicolor*	15	9	0	2	0	0	26
*Pleurotus otreatus*	18	8	1	1	0	0	29
*Schizophyllum commune*	5	10	0	1	0	1	17
*Laetiporus sulphureus*	16	7	0	6	1	0	31
*Stereum hirsutum*	18	19	1	3	0	0	43
*Agaricus bisporus*	10	6	1	1	2	0	19

Abbreviations: NRPS, nonribosomal peptides; PKS, polyketides.

**Table 6:** Putative genes involved in terpenoid backbone biosynthesis.

Pathway Map	Gene Name and Definition	EC No.	KO Term	Gene ID
map00900	FDPS; farnesyl diphosphate synthase	2.5.1.1 2.5.1.10	K00787	A03864
	GGPS1; geranylgeranyl diphosphate synthase, type III	2.5.1.1 2.5.1.10 2.5.1.29	K00804	A07435; A07657; A07658; A04007
	ACAT; acetyl-CoA C-acetyltransferase	2.3.1.9	K00626	A05025; A00976
	HMGCS; hydroxymethylglutaryl-CoA synthase	2.3.3.10	K01641	A08133; A02613
	HMGCR; hydroxymethylglutaryl-CoA reductase (NADPH)	1.1.1.34	K00021	A06620
	mvaD; diphosphomevalonate decarboxylase	4.1.1.33	K01597	A08186
	idi; isopentenyl-diphosphate Delta-isomerase	5.3.3.2	K01823	A10729
	PCYOX1; prenylcysteine oxidase	1.8.3.5 1.8.3.6	K05906	A10469
	ICMT; protein-S-isoprenylcysteine O-methyltransferase	2.1.1.100	K00587	A01157
	STE24; STE24 endopeptidase	3.4.24.84	K06013	A01427
	RCE1; prenyl protein peptidase	3.4.22.-	K08658	A07032
	hexPS; hexaprenyl-diphosphate synthase	2.5.1.82 2.5.1.83	K05355	A05688
	SRT1; ditrans, polycis-polyprenyl diphosphate synthase	2.5.1.87	K11778	A07488
	FNTA; protein farnesyltransferase/geranylgeranyltransferase type-1 subunit alpha	2.5.1.58 2.5.1.59	K05955	A02251
map00909	SQLE, ERG1; squalene monooxygenase	1.14.14.17	K00511	A10725; A02201; A02834; A03189
	FDFT1; farnesyl-diphosphate farnesyltransferase	2.5.1.21	K00801	A03300
map00130	ARO8; romatic amino acid aminotransferase I/2-aminoadipate transaminase	2.6.1.57 2.6.1.39 2.6.1.27 2.6.1.5	K00838	A06073; A01015
	wrbA; NAD(P)H dehydrogenase (quinone)	1.6.5.2	K03809	K03809; A06902; A07640
	COQ5; 2-methoxy-6-polyprenyl-1,4-benzoquinol methylase	2.1.1.201	K06127	A07348
	COQ2; 4-hydroxybenzoate polyprenyltransferase	2.5.1.39	K06125	A07585; A00920; A01883; A02465
	COQ6; ubiquinone biosynthesis monooxygenase Coq6	1.14.13.-	K06126	A02972

## Data Availability

The *S. bovinus* SB11 genome and annotations sets are available at the National Genomics Data Center, China National Center for Bioinformation, under BioProject ID PRJNA1065574.

## Supplementary Materials

The following supporting information can be downloaded at: https://www.mdpi.com/article/10.3390/jof10030211/s1, Figure S1: Hi–C contact heatmap for *S. bovinus*; Figure S2: The circular genome diagram of *S. bovinus*. Figure S3: Terpenoid biosynthesis pathway of *S. bovinus*; Figure S4: Sesquiterpenoid and triterpenoid biosynthesis pathways of *S. bovinus*; Figure S5: Ubiquinone and other terpenoid quinone biosynthesis pathways of *S. bovinus*. Table S1: Fungi and the origin of their genomes used in the study title; Table S2: Statistics of the sequencing data of the *S. bovinus;* Table S3: BUSCO assessment results of *S. bovinus*; Table S4: Summary of the annotations of *S. bovinus*; Table S5: Distribution of CAZymes in *S. bovinus* and the other 14 fungi.
