# Supplementary material for: Chromosome-Level Assembly and Comparative Genomic Analysis of Suillus bovinus Provides Insights into the Mechanism of Mycorrhizal Symbiosis
Source: J Fungi (Basel). 2024 Mar 13;10(3):211. doi: 10.3390/jof10030211 (PMC10971629; doi:10.3390/jof10030211)
Supplement: Supplementary file 1 [file jof-10-00211-s001.zip › Figure S4.pdf]

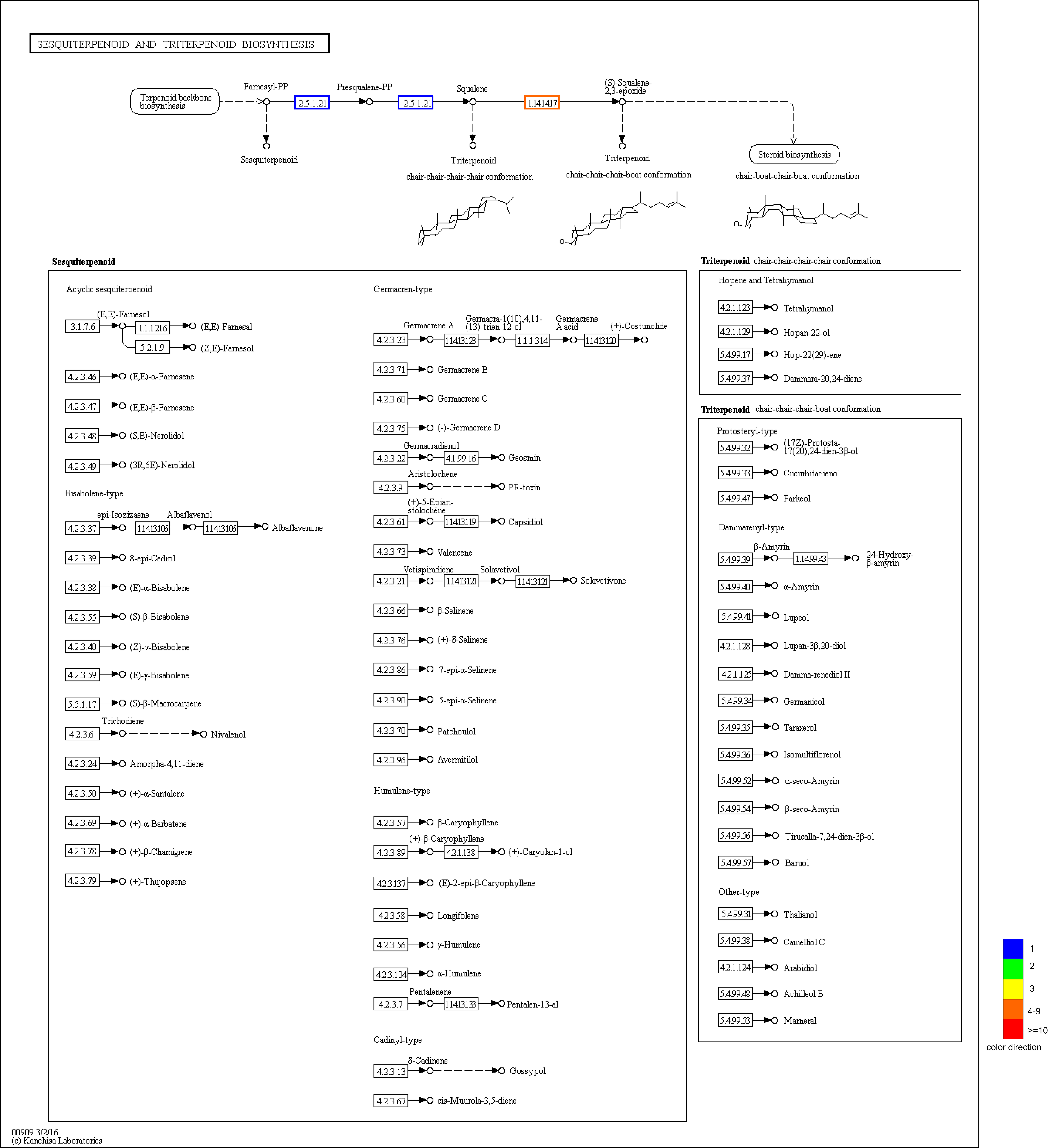
SESQUITERPENOID AND TRITERPENOID BIOSYNTHESIS
Terpenoid backbone biosynthesis
Farnesyl-PP
2.5.1.21
Presqualene-PP
2.5.1.21
Squalene
1.14.14.17
(S)-Squalene-2,3-epoxide
Sesquiterpenoid
Triterpenoid
Triterpenoid
Steroid biosynthesis
chair-chair-chair-chair conformation
chair-chair-chair-boat conformation
chair-boat-chair-boat conformation
Sesquiterpenoid
Acyclic sesquiterpenoid
(E,E)-Farnesol
3.1.7.6
1.1.1.216
(E,E)-Farnesal
5.2.1.9
(Z,E)-Farnesol
4.2.3.46
(E,E)-α-Farnesene
4.2.3.47
(E,E)-β-Farnesene
4.2.3.48
(S,E)-Nerolidol
4.2.3.49
(3R,6E)-Nerolidol
Bisabolene-type
epi-Isozizaene
Albaflavenol
4.2.3.37
1.14.13.106
1.14.13.106
Albaflavenone
4.2.3.39
8-epi-Cedrol
4.2.3.38
(E)-α-Bisabolene
4.2.3.55
(S)-β-Bisabolene
4.2.3.40
(Z)-γ-Bisabolene
4.2.3.59
(E)-γ-Bisabolene
5.5.1.17
(S)-β-Macrocarpene
Trichodiene
4.2.3.6
Nivalenol
4.2.3.24
Amorpha-4,11-diene
4.2.3.50
(+)-α-Santalene
4.2.3.69
(+)-α-Barbatene
4.2.3.78
(+)-β-Chamigrene
4.2.3.79
(+)-Thujopsene
Germacren-type
Germacrene A
Germacra-1(10),4,11-(13)-trien-12-ol
Germacrene A acid
(+)-Costunolide
4.2.3.23
1.14.13.123
1.1.1.314
1.14.13.120
4.2.3.71
Germacrene B
4.2.3.60
Germacrene C
4.2.3.75
(-)-Germacrene D
Germacradienol
4.2.3.22
4.1.99.16
Geosmin
Aristolochene
4.2.3.9
PR-toxin
(+)-5-Epiaristolochene
4.2.3.61
1.14.13.119
Capsidiol
4.2.3.73
Valencene
Vetispiradiene
Solavetivol
4.2.3.21
1.14.13.121
1.14.13.121
Solavetivone
4.2.3.66
β-Selinene
4.2.3.76
(+)-δ-Selinene
4.2.3.86
7-epi-α-Selinene
4.2.3.90
5-epi-α-Selinene
4.2.3.70
Patchoulol
4.2.3.96
Avermitilol
Humulene-type
4.2.3.57
β-Caryophyllene
(+)-β-Caryophyllene
4.2.3.89
4.2.1.138
(+)-Caryolan-1-ol
4.2.3.137
(E)-2-epi-β-Caryophyllene
4.2.3.58
Longifolene
4.2.3.56
γ-Humulene
4.2.3.104
α-Humulene
Pentalenene
4.2.3.7
1.14.13.133
Pentalen-13-al
Cadinyl-type
δ-Cadinene
4.2.3.13
Gossypol
4.2.3.67
cis-Muurola-3,5-diene
Triterpenoid chair-chair-chair-chair conformation
Hopene and Tetrahymanol
4.2.1.123
Tetrahymanol
4.2.1.129
Hopan-22-ol
5.4.99.17
Hop-22(29)-ene
5.4.99.37
Dammara-20,24-diene
Triterpenoid chair-chair-chair-boat conformation
Protosteryl-type
5.4.99.32
(17Z)-Protosta-17(20),24-dien-3β-ol
5.4.99.33
Cucurbitadienol
5.4.99.47
Parkeol
Dammarenyl-type
β-Amyrin
5.4.99.39
1.14.99.43
24-Hydroxy-β-amyrin
5.4.99.40
α-Amyrin
5.4.99.41
Lupeol
4.2.1.128
Lupan-3β,20-diol
4.2.1.125
Damma-renediol II
5.4.99.34
Germanicol
5.4.99.35
Taraxerol
5.4.99.36
Isomultiflorenol
5.4.99.52
α-seco-Amyrin
5.4.99.54
β-seco-Amyrin
5.4.99.56
Tirucalla-7,24-dien-3β-ol
5.4.99.57
Baruol
Other-type
5.4.99.31
Thalianol
5.4.99.38
Camelliol C
4.2.1.124
Arabidiol
5.4.99.48
Achilleol B
5.4.99.53
Marneral
00909 3/2/16
(c) Kanehisa Laboratories
1
2
3
4-9
>=10
color direction
