# Supplementary material for: Chromosome-Level Assembly and Comparative Genomic Analysis of Suillus bovinus Provides Insights into the Mechanism of Mycorrhizal Symbiosis
Source: J Fungi (Basel). 2024 Mar 13;10(3):211. doi: 10.3390/jof10030211 (PMC10971629; doi:10.3390/jof10030211)
Supplement: Supplementary file 1 [file jof-10-00211-s001.zip › Supplemental Materials.pdf]

Chromosome-Level Assembly and Comparative Genomic Analysis of *Suillus bovinus* Provides Insights into the Mechanism of Mycorrhizal Symbiosis

Jinhua Zhang [1,2], Mengya An [1], Yanliu Chen [1], Shengkun Wang [1], Junfeng Liang [1,*]

1 Research Institute of Tropical Forestry, Chinese Academy of Forestry, Guangzhou 510520, China

2 College of Forestry, Nanjing Forestry University, Nanjing 210037, China

* Corresponding author:

Junfeng Liang, E-mail: jfliang2000@163.com.

Mailing address: Research Institute of Tropical Forestry, Chinese Academy of Forestry, Guangzhou 510520, China.

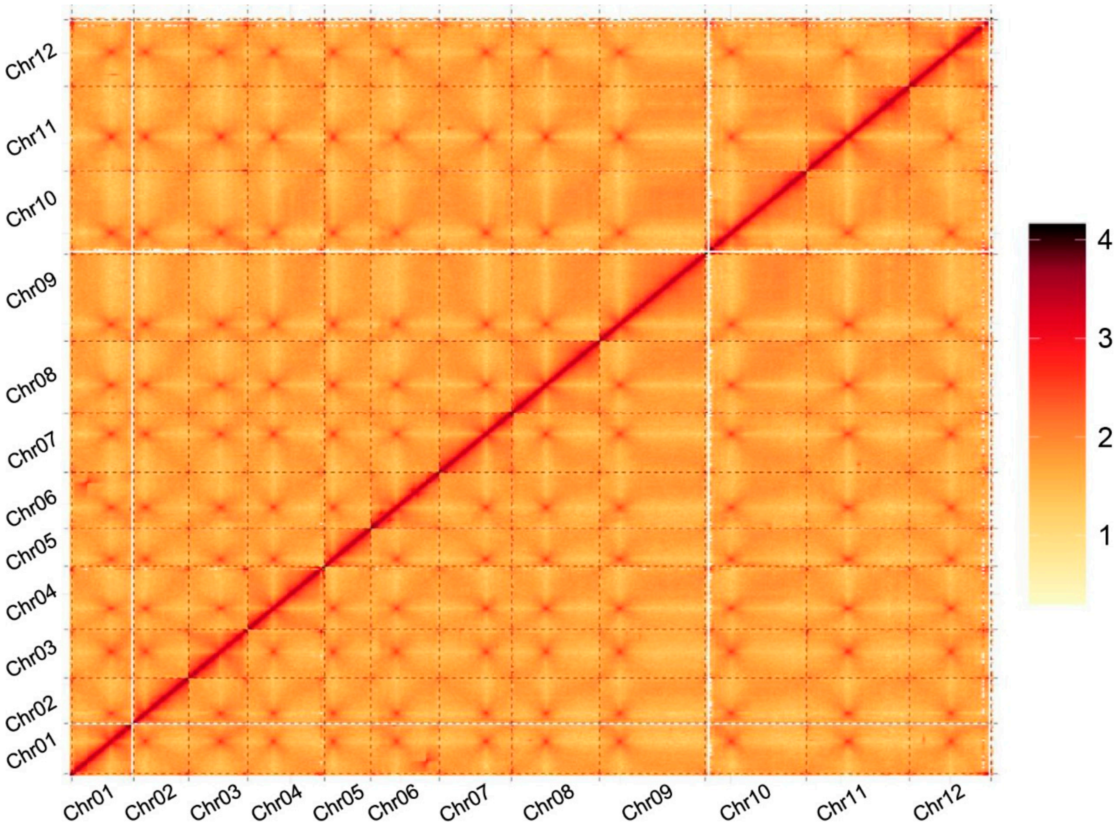

Figure S1. Hi–C contact heatmap for *S. bovinus*.

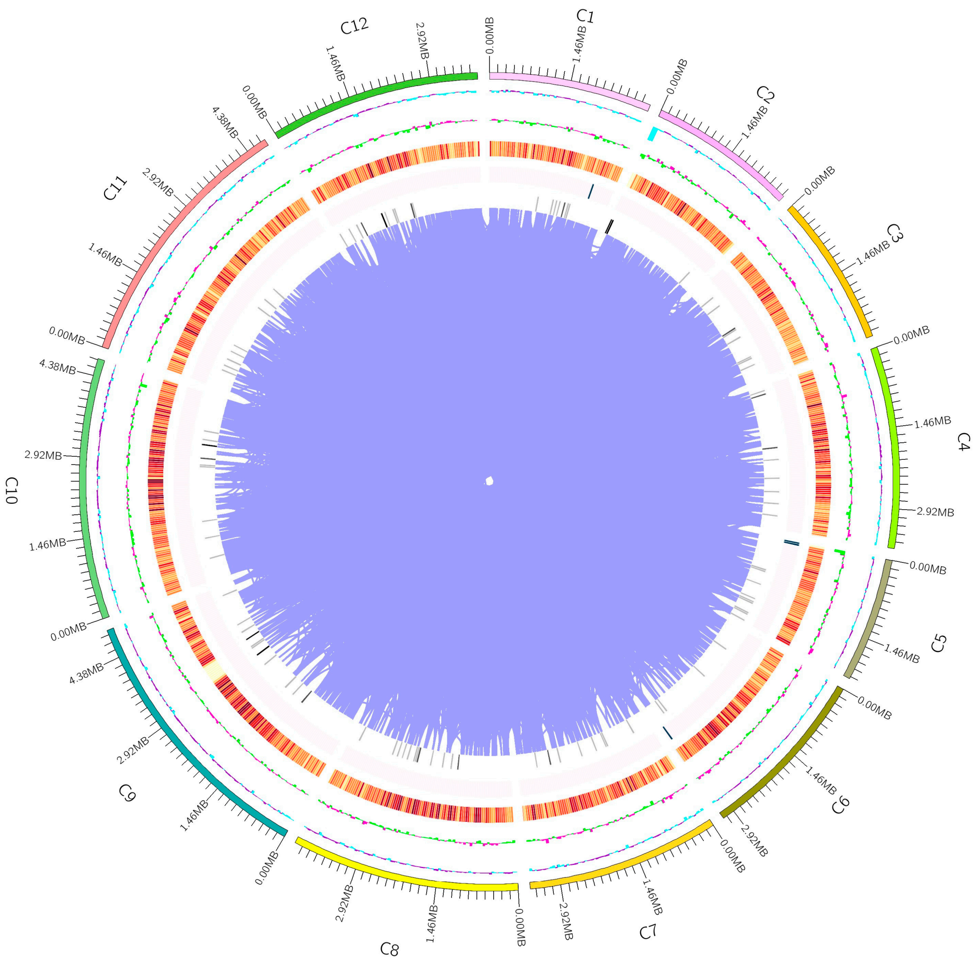

Figure S2. The circular genome diagram of *S. bovinus*.

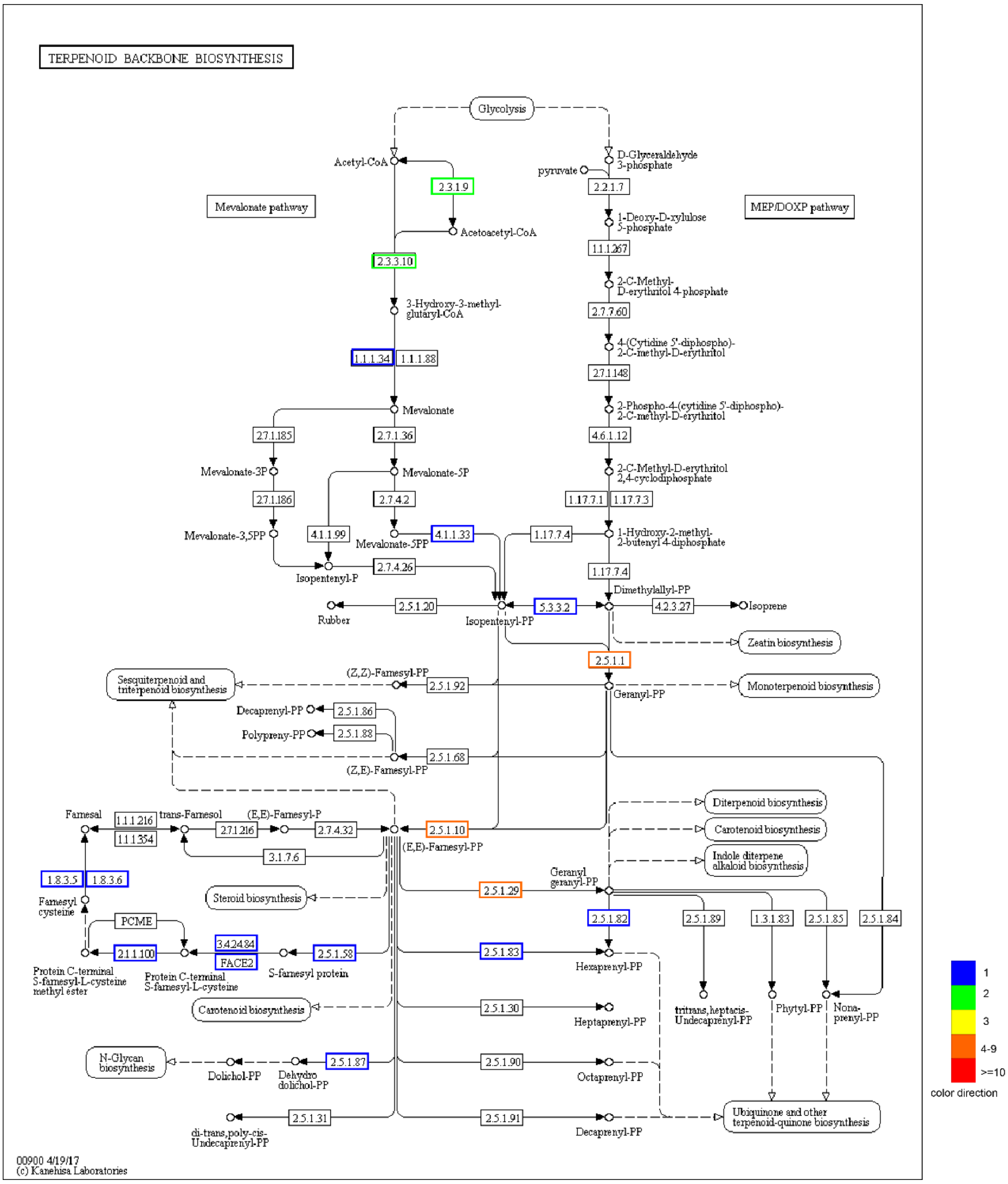

Figure S3. Terpenoid biosynthesis pathway of *S. bovinus*.

Note: The different colors of the boxes indicate the number of corresponding genes. Blue represents 1 gene, green represents 2 genes, yellow 4-9 genes, and red represents more than 10 genes, while white means not.

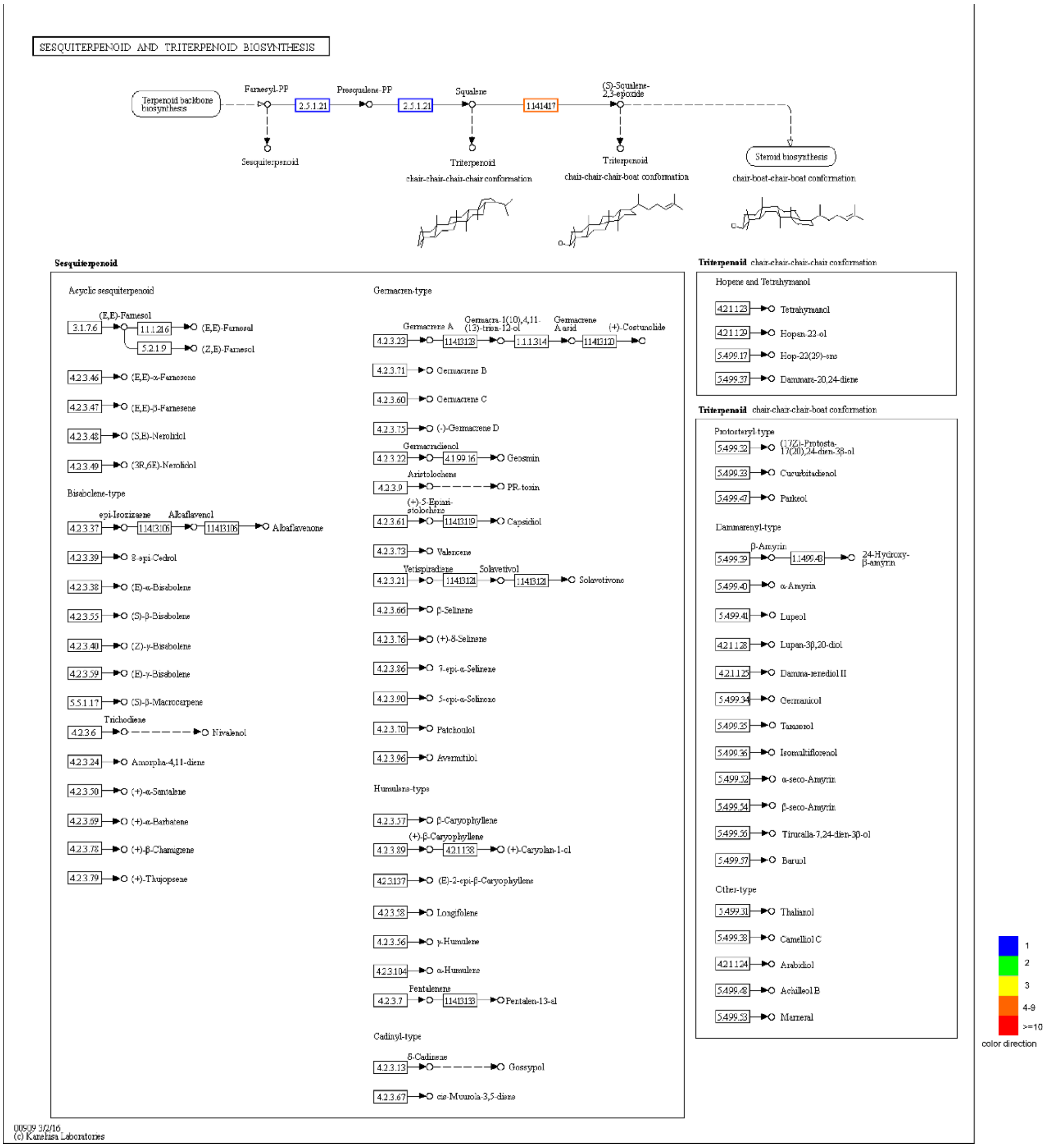

Figure S4. Sesquiterpenoid and triterpenoid biosynthesis pathway of *S. bovinus*.

Note: The different colors of the boxes indicate the number of corresponding genes. Blue represents 1 gene, green represents 2 genes, yellow 4-9 genes, and red represents more than 10 genes, while white means not.

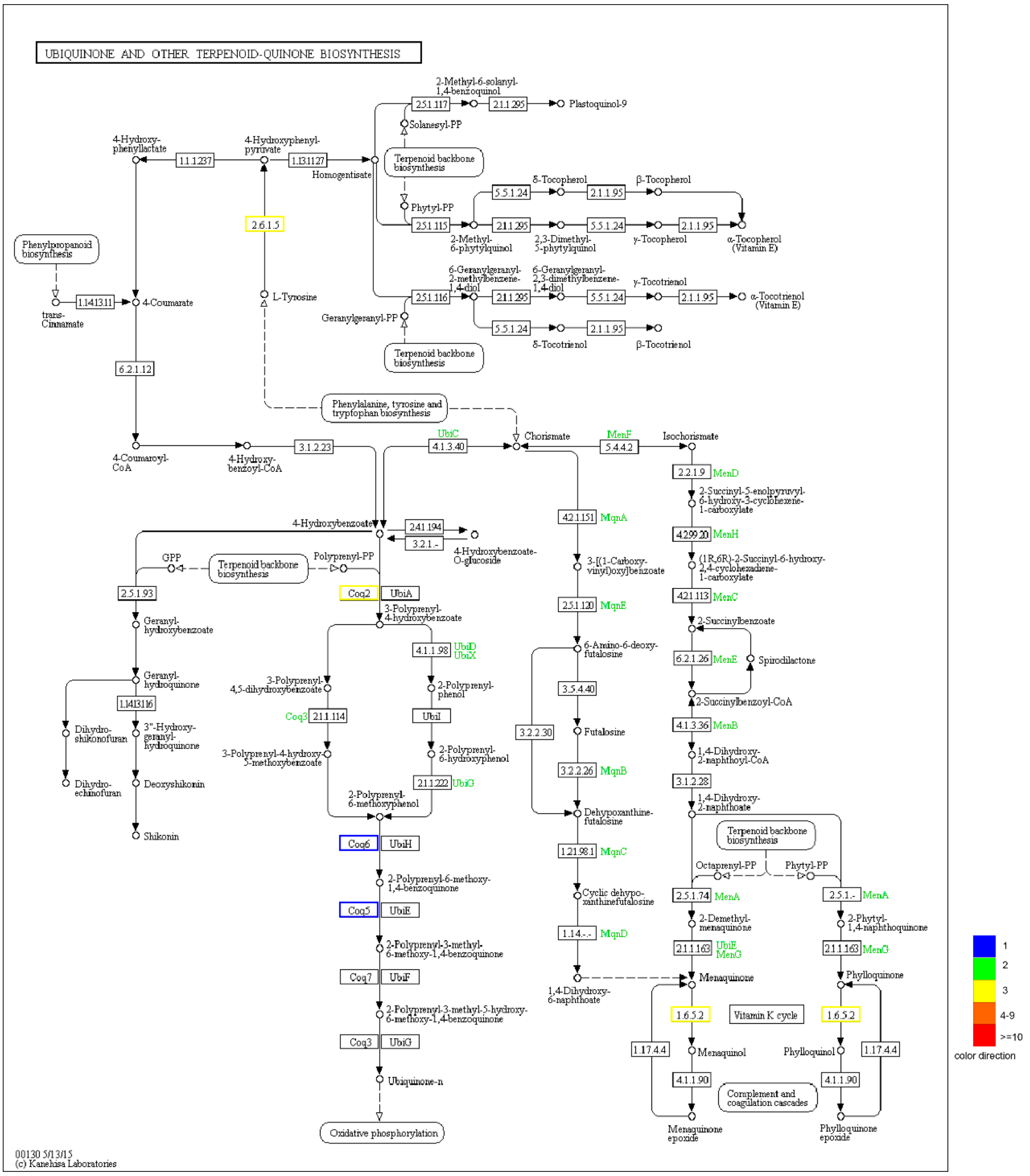

Figure S5. Ubiquinone and other terpenoid quinone biosynthesis pathway of *S. bovinus*.

Note: The different colors of the boxes indicate the number of corresponding genes. Blue represents 1 gene, green represents 2 genes, yellow 4-9 genes, and red represents more than 10 genes, while white means not.
