# Supplementary figures and images for: Chromosome-Level Assembly and Comparative Genomic Analysis of Suillus bovinus Provides Insights into the Mechanism of Mycorrhizal Symbiosis
Source: J Fungi (Basel). 2024 Mar 13;10(3):211. doi: 10.3390/jof10030211 (PMC10971629; doi:10.3390/jof10030211)

TERPENOID BACKBONE BIOSYNTHESIS

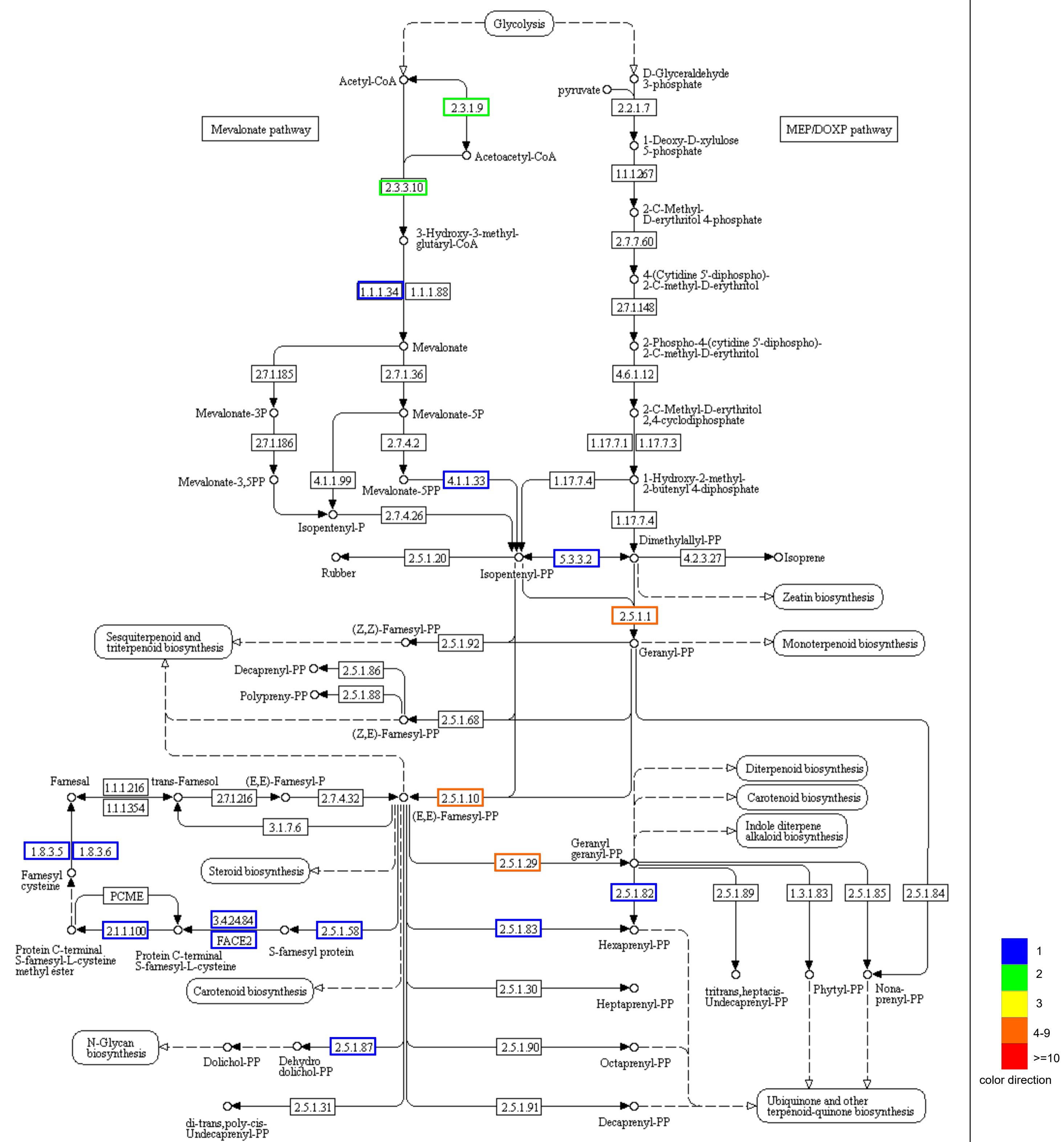

Supplement: Supplementary file 1 [file jof-10-00211-s001.zip › Figure S3.pdf]

UBIQUINONE AND OTHER TERPENOID-QUINONE BIOSYNTHESIS

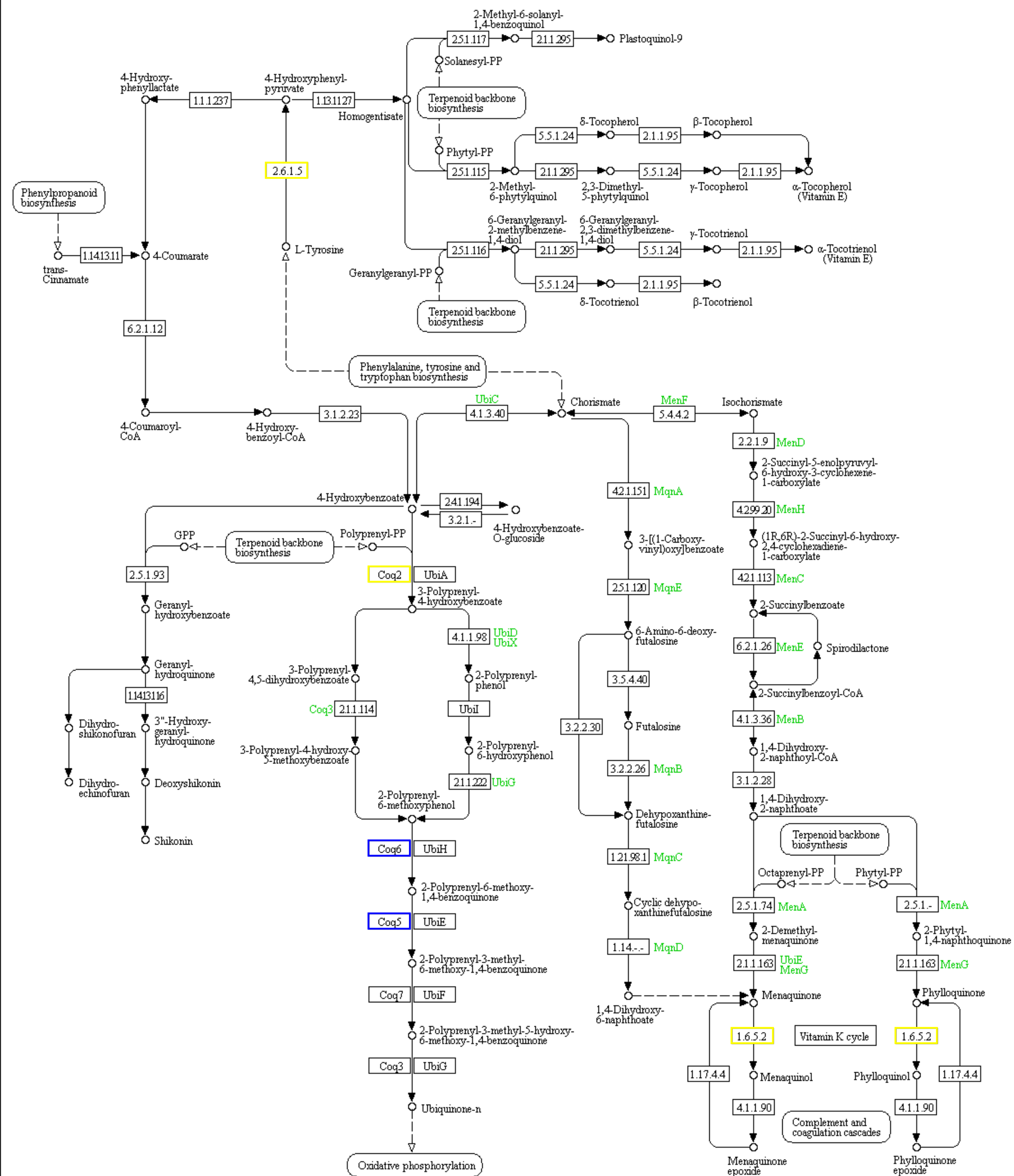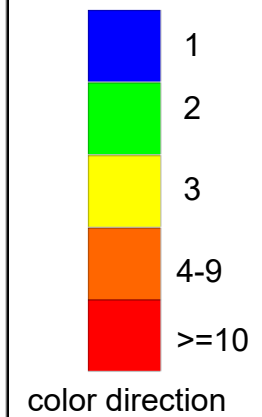

Supplement: Supplementary file 1 [file jof-10-00211-s001.zip › Figure S5.pdf]

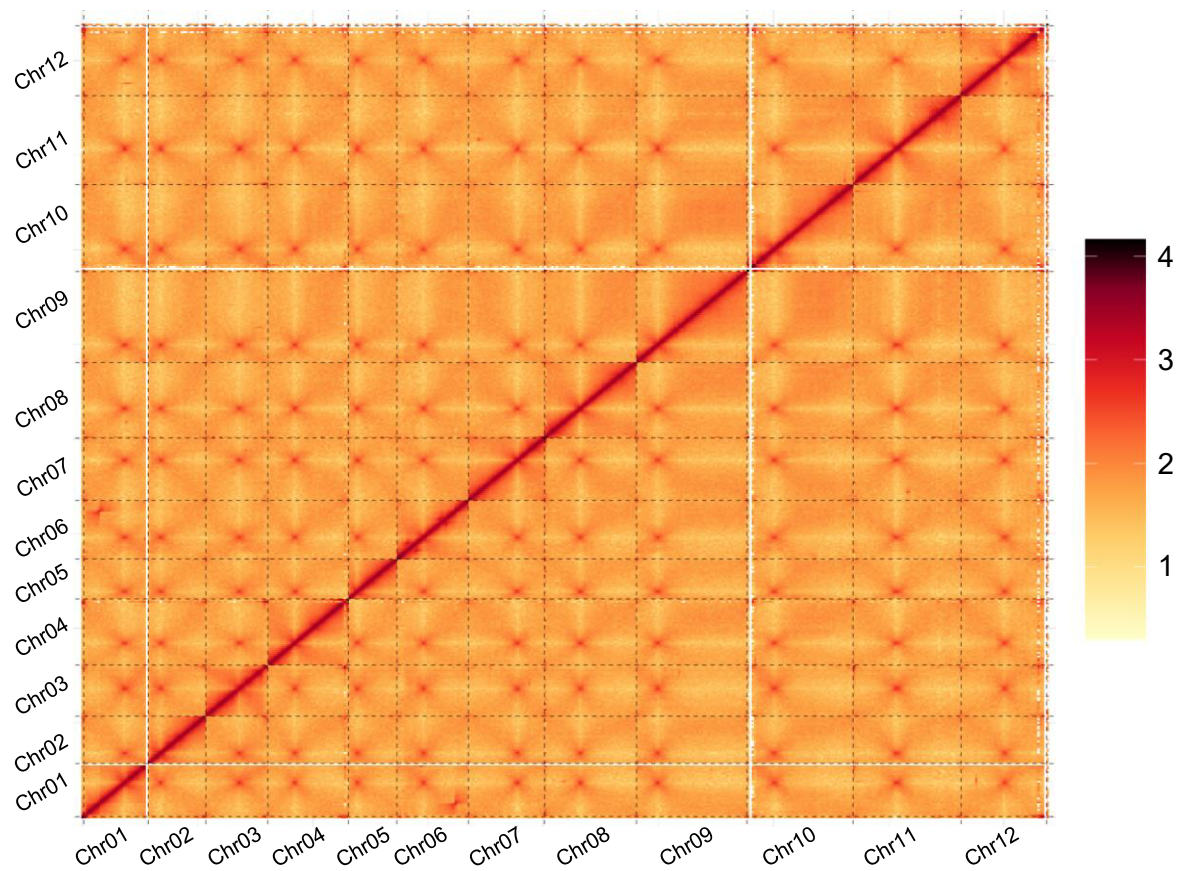

Supplement: Supplementary file 1 [file jof-10-00211-s001.zip › Figure S1.pdf]
